# Comparison between Histological Features and Strain Elastographic Characteristics in Canine Mammary Carcinomas

**DOI:** 10.3390/vetsci9010009

**Published:** 2021-12-28

**Authors:** Nieves Pastor, Lorena Espadas, Massimo Santella, Luis Javier Ezquerra, Raquel Tarazona, María Esther Durán

**Affiliations:** 1 Veterinary Teaching Hospital, University of Extremadura, 10003 Cáceres, Spain; nievespastor@unex.es (N.P.); lorenaeg@unex.es (L.E.); massimo.santella@gmail.com (M.S.); 2 Animal Medicine and Surgery Unit, Department of Animal Medicine, Faculty of Veterinary Medicine, University of Extremadura, 10003 Cáceres, Spain; 3 Immunology Unit, Department of Physiology, Faculty of Veterinary Medicine, University of Extremadura, 10003 Cáceres, Spain; 4 Anatomy and Comparative Pathological Anatomy, Department of Animal Medicine, Faculty of Veterinary Medicine, University of Extremadura, 10003 Cáceres, Spain; esther@unex.es

**Keywords:** strain elastography, mammary tumors, strain ratio, color scale, histological features

## Abstract

Elastography is a sonographic technique that provides a noninvasive evaluation of the stiffness of a lesion. The objective of this work was to evaluate the accuracy of strain elastography, the most accessible modality in clinical practice, to discriminate between different histological types of malignant mammary neoplasms in the canine species, which can provide complementary information in real time to the diagnosis and thus help in the choice of surgical technique. A total of 34 females with 56 mammary carcinomas were selected and classified into three histological groups according to their aggressiveness. The histological and elastographic characteristics of these malignant tumors were analyzed and compared to evaluate the diagnostic accuracy of strain elastography. Visual score presented a sensitivity of 88.0%, specificity of 58.1%, and accuracy of 71.43% in distinguishing the most aggressive group of carcinomas. The strain ratio had a sensitivity of 84.0%, specificity of 61.1%, and accuracy of 69.64%. On the other hand, intratumoral strain ratio obtained a sensitivity of 71.40% and specificity of 61.90% when intratumoral fibrosis was taken as reference, with an accuracy of 66.07%. Similarly, peritumoral strain ratio was also positively related to fibrosis in the periphery of lesions (*p* ≤ 0.001), with a sensitivity of 93.80%, specificity of 77.50% and an accuracy of 92.87%. In conclusion, accuracy of this elastographic modality can be a useful method to differentiate more aggressive histological types. Therefore, it represents an additional diagnostic technique useful in the daily clinic thanks to the short time required for the examination, which allows real-time visualization and immediate interpretation of the results.

## 1. Introduction

Canine mammary cancer has great clinical relevance due to their high prevalence and mortality, which has been shown to be a valid model for the breast cancer in women [1,2]. An increase in the diagnosis of malignant tumors has recently been described up to 70% [3,4], 80% [5,6], and even 88.13% [7].

Daily, clinical veterinarians assist patients with mammary tumors, establishing their diagnosis, prognosis, and treatment [8]. Use of precise pre-surgical diagnostic techniques that allow for the differentiation of these tumors is a clinical strategy with great diagnostic, prognostic, and therapeutic value in patients with malignant mammary neoplasms [9]. The heterogeneity present in mammary tumors, given the diversity of cellular components, the presence of necrosis, the irregular distribution of the lesions, and even the puncture in inappropriate sites, justifies that methods such as cytology have a highly variable accuracy, so it is necessary to establish complementary diagnostic methods [10].

Tumor size is considered as one of the main variables (along with the disease extent, lymphatic drainage, location of the tumor, etc.) to be taken into consideration to define the correct therapeutic approach [11]. Thus, surgical approach and disease prognosis vary between the different types of tumors and the histological grade of malignancy, requiring a more precise clinical evaluation of lesions, especially those of small size [6].

Ultrasound is a non-invasive technique that allows real-time evaluation of breast tissue, playing an essential role in human medicine as a complementary method to mammography [12]. However, in the canine species, the literature is not consistent in its predictive value to discriminate between benign and malignant lesions [13,14,15].

Elastography has been introduced in both human and veterinary medicine as a tool to classify the nature of tumor lesions, suggesting malignancy if a decrease in plasticity is observed [16,17]. There are currently two types of elastographic techniques: shear wave elastography and strain elastography [18].

Shear wave elastography provides quantitative information of the stiffness of tissues by measuring the propagation speed of shear waves generated by acoustic radiation force in kilopascals or meters per second [19]. In contrast, strain elastography is a real-time technique that estimates tissue stiffness according to the strain ratio, evaluating tissue elasticity by comparing local tissue displacement before and after applying a force of compression of the transducer [20]. However, the measurements obtained with this strain elastographic technique are not directly quantifiable, so several qualitative and semi-quantitative methods have been proposed [21].

Therefore, the objective of this work was to evaluate the accuracy of strain elastography to discriminate, by means of these qualitative and semi-quantitative methods, between different histological types of malignant mammary neoplasms in the canine species, to provide complementary information in real time to conventional diagnosis, in addition to helping in the choice of surgical technique. Having additional information on the aggressiveness of the tumors prior to surgical management can be especially important in cases of large patients with concomitant diseases that affect anesthetic risk and in which intervention requires a long time, or even owners that do not accept radical aggressive surgeries in cases with very small tumors.

## 2. Materials and Methods

### 2.1. Subjects of Study

A prospective study was carried out in 34 female dogs diagnosed with malignant mammary tumors at the Veterinary Teaching Hospital of the University of Extremadura (Cáceres, Spain). Informed consent was obtained from the owners prior to the animals being included in this study. Only mammary lesions from 0.5 cm to 3 cm were included in this study. The patients underwent an ultrasound examination prior to surgery and did not undergo any procedure other than those necessary for the diagnosis and treatment of the disease. A single patient who presented a benign tumor, when performing the histological study, was excluded from the study.

### 2.2. Ultrasound Elastography and Analysis

The ultrasound examination was carried out by a single sonographer using the Logiq S7 Expert ultrasound machine (GE Healthcare^®^, Madrid, Spain) with a linear transducer with a frequency range of 4.5–12 MHz, using the elastography module.

First, under general anesthesia of the patients, a first B-mode ultrasound examination was performed to then generate elastographic images by applying light repeated compressions perpendicular to the tumor and the entire adjacent region with the probe. To avoid insufficient or excessive tissue pressure, the correct pressure exerted by the probe on the lesion has been determined, thanks to a vertical column on the ultrasound monitor that shows a green color when the pressure exerted is correct, yellow when a partial pressure is applied, and a red color if it is inappropriate. Subsequently, three images of each tumor were taken to visualize the tumor in different ultrasound slices and calculate the mean strain ratio.

The evaluation of the elastograms was carried out using both a qualitative (visual scale) and a semi-quantitative method (strain-ratio).

For the qualitative analysis of the elastography, a visual color scale was used in which the most compressible tissues appeared in red, those of intermediate stiffness in green, while the least compressible defined in blue. According to the criteria of Itoh et al. [22], lesions were distributed into five categories, according to which lesions classified with a score of 1, 2, and 3 are considered as probably benign, while probably malignant lesions were classified with a score of 4 and 5.

For the semi-quantitative analysis, a ratio was used that compares the rate of deformability between two manually selected areas of interest (strain ratio), the compressibility within the lesion was compared with that of the adjacent healthy subcutaneous tissue. This proportion reflects the relative rigidity of the lesion, which is directly proportional to the probability of malignancy [23,24].

### 2.3. Histological Analysis

Following the ultrasonographic examination, a mastectomy was carried out for the excision of the mammary masses, surgical removal, and subsequent referral of the biopsies to the pathology service of our hospital for histopathological study. All biopsies were analyzed by the same pathologist unknowing elastographic data. To assess the degree of fibrosis of the lesions, Masson’s Trichrome stain was performed in addition to the Hematoxylin-Eosin stain.

The neoplasms were classified using the histological classification of Zapulli et al. [25]. The tumors were then grouped into three categories taking into account our previous work in which we analyzed the aggressiveness and prognosis of canine mammary carcinomas classified according to histological diagnosis [26]. Based on these results, we established the following three categories: Group a—complex carcinoma; Group b—mixed carcinoma/ductal-papillary intraductal carcinoma; and Group c—simple carcinoma/anaplastic-inflammatory carcinoma/carcinosarcoma/adenosquamous carcinoma/other special types of carcinomas. In addition to diagnosis, the histological grade of malignancy [27], microscopic necrosis (evaluated as the presence or absence and classified as low, intermediate, and high according to the number of necrosis foci and the extent of the necrosis) as well as peritumoral and intratumoral fibrosis were graded as mild–moderate and intense. The presence of collagen, both from the point of view of its extension and the intensity of fibrosis, was compared with the non-tumor breast tissue adjacent to the lesion. Finally, patients were classified according to their clinical stage [28].

### 2.4. Statistical Analysis

Statistical analysis was performed with the Statistical Package for the Social Science version 22.0 software (SPSS, Chicago, IL, USA). The normality of the variables was tested using the Kolmogorov–Smirnov test.

A descriptive analysis of all the epidemiological, histological, and elastographic variables was carried out. The Pearson chi square was used to determine the relationship between qualitative elastographic variables and histological variables. On the other hand, the Mann–Whitney U and the Kruskal–Wallis tests were used to compare the qualitative and quantitative elastographic and histological variables. A *p* value of less than 0.05 was considered significant.

To assess the diagnostic validity of elastography, histology was considered the gold standard test. Diagnostic performance parameters including sensitivity, specificity, area under the curve (AUC), optimal cut-off values, and accuracy were calculated using receiver-operating characteristic curve (ROC) analysis in a logistic regression model.

## 3. Results

### 3.1. Study Sample

We studied 34 female dogs diagnosed with malignant mammary tumors with a total of 56 malignant mammary neoplasms analyzed. The mean age of the patients included in this study was 10.36 ± 2.84 (mean ± standard deviation [SD]) years (interquartile range [IQR] = 4 years). The patients belonged to the following breeds: mixed (26.47%; n = 9), Labrador retriever (11.76%; n = 4), Yorkshire terrier (8.82%; n = 3), Breton Spaniel (5.88%, n = 2), Coker spaniel (5.88%, n = 2), German shepherds (5.88%, n = 2), Mastiffs (5.88%, n = 2), German pointer (5.88%, n = 2), Maltese Bichon (2.94%, n = 1), Spanish greyhound (2.94%, n = 1), Prague mouse (2.94%, n = 1), Boxer (2.94%, n = 1), Fox terrier (2.94%, n = 1), Border collie (2.94%, n = 1), Siberian husky (2.94%, n = 1), and Miniature Pincher (2.94%, n = 1).

Thirty-two malignant tumors were found at the left mammary chain (57.14%) and 24 at the right chain (42.86%). Two tumors (3.57%) were located in the first mammary gland, 5 (8.93%) in the second, 10 (17.86%) in the third, 15 (26.78%) in the fourth, and 24 (42.86%) in the fifth mammary gland.

Regarding the reproductive status of the patients, only 14.71% (n = 5) were spayed at the time of diagnosis.

After evaluation, the patients’ clinical stage prior surgery, it was observed that 97.1% (n = 33) of the patients were in stage I of the disease and only 2.9% (n = 1) of them presented with stage IV.

The patient with the benign tumor was a five year old intact German pointer with an isolated mammary tumor in the fourth mammary gland of the left chain.

### 3.2. Histological Study

As shown in Table 1, the histopathological examination revealed a total of 32.14% (n = 18) neoplasms of Group a, 25.0% (n = 14) neoplasms of Group b, and 42.86% (n = 24) of Group c.

Regarding the histological grade of malignancy, 39.28% (n = 22) were low grade (I) neoplasms, 55.4% (n = 31) were intermediate grade (II), and 5.5% (n = 3) showed high grade of malignancy (III).

In terms of intratumoral fibrosis, 37.5% (n = 21) of the tumors showed mild or moderate fibrosis compared to 62.5% (n = 35) in which a high degree of fibrosis was observed. On the other hand, 71.43% (n = 40) of the neoplasms exhibited mild or moderate peritumoral fibrosis, while 28.57% (n = 16) had high peritumoral fibrosis.

Finally, regarding microscopic necrosis and vascular invasion, these were only seen in 23.21% (n = 13) and 7.1% (n = 4) of the samples, respectively.

The only benign tumor diagnosed during the study period presented mild intratumoral and peritumoral fibrosis, without microscopic necrosis or vascular invasion.

### 3.3. Elastography Ultrasound

On the visual color scale, 37.5% (n = 21) of the neoplasms obtained a score of 3, 58.9% (n = 33) of 4, and 3.6% (n = 2) obtained a score of 5.

On the other hand, in the semi-quantitative analysis using the intratumoral strain ratio, a mean was obtained for all tumors of 4.38 (SD: 0.46; 95% confidence interval [CI]: 4.25, 4.51), with the mean for Group a of 4.24 (SD: 0.45; 95% CI: 4.05, 4.43), for group b of 4.19 (SD: 0.38; 95% CI: 3.96, 4.39), and for group c of 4.59 (SD: 0.50; 95% CI: 4.39, 4.78). The mean strain ratio calculated for all tumors in the peripheral tissues near the lesions was 3.48 (SD: 1.25; 95% CI: 2.77, 3.40). The analysis according to the tumor classification showed a mean strain ratio in the peripheral tissue that increased from Group a to c, observing a mean of 3.15 (SD: 1.18; 95% CI: 2.31, 3.99) for Group a, 3.44 (SD: 1.53, 95% CI: 2.35, 4.53) for Group b, and 3.74 (SD 1.10; 95% CI 3.11, 4.38) for Group c.

The only benign tumor diagnosed presented a score of 2 on the visual color scale and its intratumoral strain ratio was 2.3, while the peritumoral strain ratio was 2.5.

### 3.4. Associations between Histological and Elastography Variables

**Figure 1:**
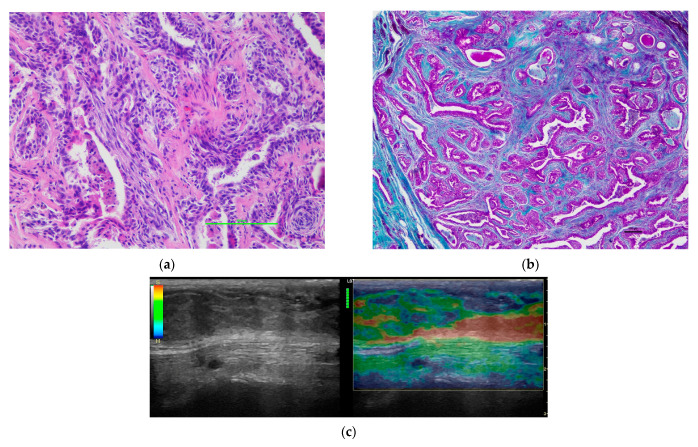
Representative image of a mass confirmed as a complex carcinoma. (**a**) Detail of the cellular composition of the tumoral tissue (H&E). (**b**) Histological detail of the tumor parenchyma of a complex carcinoma in which there was low presence of collagen fibers (Masson’s Trichrome). (**c**) B-mode and elastographic images scored as 3 in the visual scale, corresponding to the same complex carcinoma sample.

**Figure 2:**
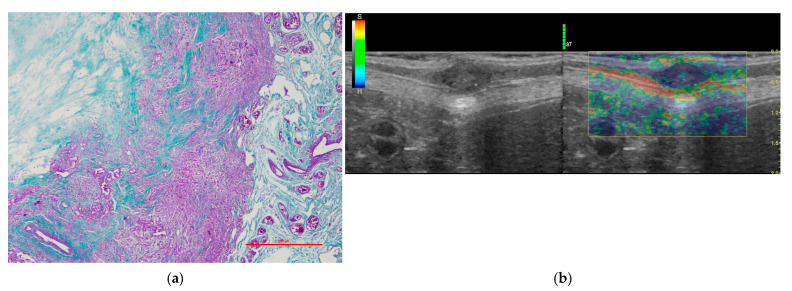
Representative image of a mass confirmed as an anaplastic carcinoma. (**a**) Histological detail of an anaplastic carcinoma with wide areas of fibrosis (Masson´s Trichrome). (**b**) B-mode and elastographic images scored as 4 in the visual scale, corresponding to the same anaplastic carcinoma sample.

**Figure 3:**
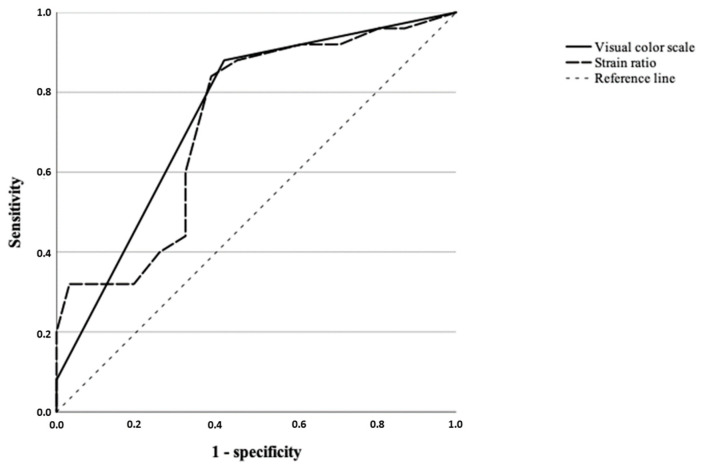
Receiver-operating characteristic curve for the visual color scale (qualitative analysis) (*p* = 0.002) and for the strain ratio (semiquantitative analysis) (*p* = 0.004).

Statistically significant differences were obtained in the visual color scale (qualitative analysis) among the different histological diagnosis (*p* = 0.013). We observed a score of 3 in the 55.55% of tumors (n = 10) in group a (Figure 1), while 81.81% (n = 18) of tumors in Group c were classified with a score of 4 (Figure 2). Regarding the histological diagnosis, the ROC curve analysis for the visual color scale showed a sensitivity of 88.0% and a specificity of 58.1% with an AUC of 0.747 (Figure 3). The accuracy of this test (visual color scale) was 69.64% to distinguish the patients in Group c with respect to the rest of the histological diagnostic groups. No further associations were observed between the visual color scale and other histological variables.

Regarding the semi-quantitative analysis, statistically significant differences were obtained when the strain ratio and the histological diagnosis were compared, observing how the mean for the intratumoral strain ratio of Group a (4.24) was significantly lower than that of Group c of diagnosis (4.59) (*p* = 0.018); obtaining a sensitivity of 84.0%, specificity of 61.1%, and area under the curve of 0.726, with a cut-off point of 4.25 and accuracy of 71.43% (Figure 3).

**Figure 4:**
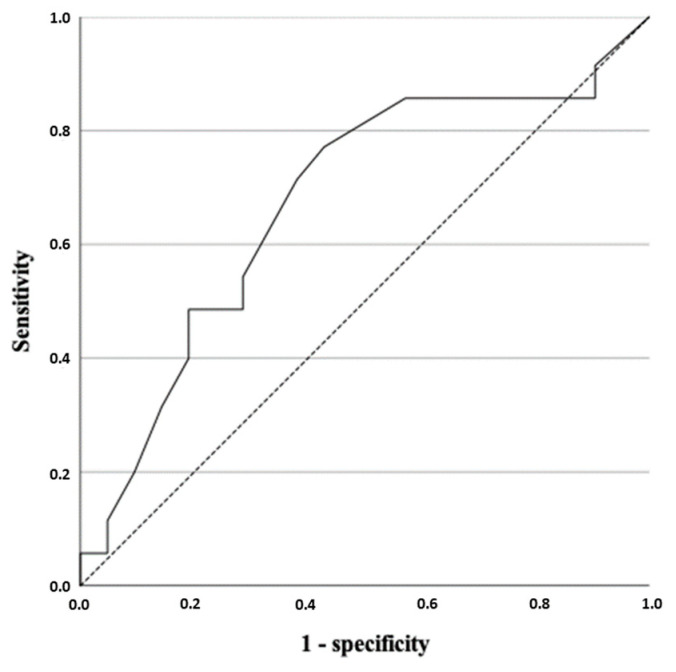
Receiver-operating characteristic curve for the strain ratio (semiquantitative analysis) taking intratumoral fibrosis as the reference (*p* = 0.03).

**Figure 5:**
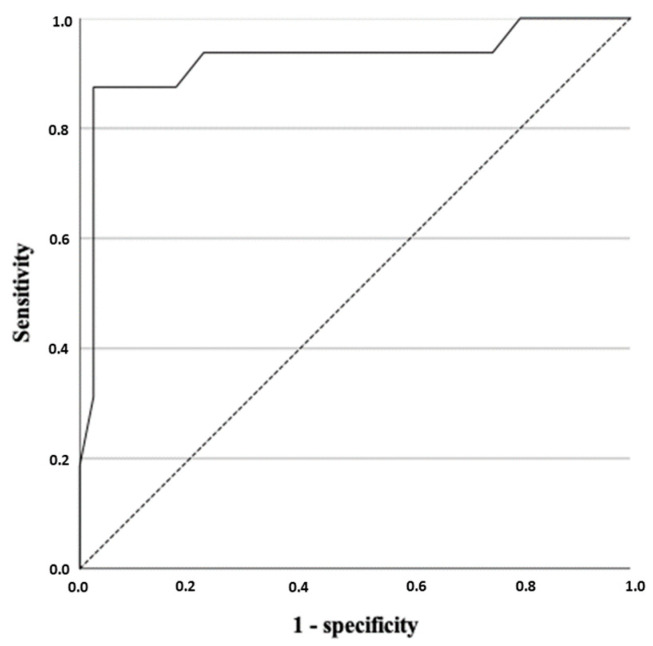
Receiver-operating characteristic curve for the peritumoral strain ratio (semiquantitative analysis) taking peritumoral fibrosis as the reference (*p* ≤ 0.001).

The intratumoral strain ratio was higher in those neoplasms with intratumoral fibrosis (*p* = 0.03). The ROC curve analysis showed that the best cut-off for the intratumoral strain ratio was 4.25, with a sensitivity of 71.40%, a specificity of 61.90%, an AUC of 0.674 (Figure 4) and an accuracy of 66.07%. The peritumoral strain ratio was also higher in those neoplasms with peritumoral fibrosis (*p* ≤ 0.001). The analysis of the ROC curve for the peritumoral strain ratio allowed us to establish a cut-off point of 2.95, with a sensitivity of 93.80%, a specificity of 77.50%, and an AUC of 0.923 with an accuracy of 92.86% (Figure 5).

No association was found between the strain ratio and the histological grade of malignancy or with tumor necrosis.

We observed in two tumors of Group c (mucinous carcinoma and lipid rich carcinoma) areas of central necrosis that gave a result of low strain ratios and therefore may represent false negatives. We also observed a high degree of fibrosis in one case of complex carcinoma (group a) that resulted in a high strain ratio.

The results for the elastographic variables are summarized in Table 2, Table 3 and Table 4.

## 4. Discussion

There are few works in the veterinary literature that demonstrate the usefulness of elastography for the evaluation of mammary tumors in the canine species. Most of these studies are based on the use of this ultrasound technique to predict the malignancy of the lesions [17,29,30,31]. To our knowledge there is only one study that compares the precision of several ultrasound methods in the identification of different types of malignant mammary carcinomas in female dogs [32]. On the other hand, all of these studies used quantitative elastographic methods, which have greater precision and reproducibility than strain elastography, but have a higher economic cost.

In this work, we analyzed the efficacy of strain elastography for the study of mammary tumors of the canine species. Therefore, the main objective of this work was to evaluate the validity of this elastographic modality in different histological types of malignant mammary neoplasms in the canine species. This technique, strain elastography, can represent a complementary diagnostic tool that veterinary clinics can access more easily due to its lower economic cost compared to other types of elastographic modalities.

Strain elastography is an ultrasound technique that allows for the estimation of the stiffness of tissues by simulating manual palpation and successfully differentiating the nature of mammary lesions [22,33,34]. In our study, the elastogram characteristics were those expected, corroborating the malignancy of the tumors diagnosed by histology. These characteristics are similar to those described in women since they present a predominance of blue tones both in the visual color scale [22,34,35,36,37] as well as observing a high strain ratio [24,34,35,36]. In our work, we have not been able to make a comparison between benign and malignant mammary tumors because only one benign tumor was diagnosed compared to the 56 malignant neoplasms included in the study. However, the elastographic characteristics of this tumor were similar to those described in the rest of the studies [24,34,35,36], clearly differentiating from the rest of the samples.

Our results agreed with Zhou et al. (2013) [37], showing a higher strain ratio in the peritumoral tissue of those tumors with more aggressive histological features (Group c), supporting previous studies that describe an increase in stiffness of the peripheral tissue close to the lesion in malignant neoplasms [22,38,39], probably due to cancer cell infiltration in the peripheral tissue [22]. In our work, an increase in the intratumoral strain ratio was also observed in those tumors with a higher degree of fibrosis, results that agree with those of the previously mentioned studies [19,32,34]. Furthermore, the evaluation of the perilesional tissue could serve as a reference to distinguish false negatives when analyzing benign tumors with greater stiffness such as fibroadenomas [11]. In our hands, the high diagnostic value of the peritumoral elastographic study (92.86%) with respect to peritumoral fibrosis, supports its value as a very useful and affordable diagnostic technique that can guide the veterinary clinician on the aggressiveness of the tumor.

On the other hand, strain elastography did not show significant differences according to the degree of malignancy, in accordance with the results of Feliciano et al. (2018) [28]. In our study, the small number of patients with high grade of malignancy (n = 3) made the statistical comparison between groups difficult.

Regarding the validity of strain elastography as a diagnostic test to discriminate between different histopathological diagnosis groups, we obtained a sensitivity and specificity of 84.0%, and 61.1% for diagnosing Group c of carcinomas with an accuracy of 69.64% by semi-quantitative analysis (strain ratio); this accuracy was higher when using qualitative methods (71.43%) with a sensitivity and specificity of 88.0%, and 58.1%, respectively. Our results agreed with those of Feliciano et al. (2018), who obtained an accuracy of 75% [28]. Although the accuracy of strain elastography is limited to differentiating between different types of mammary carcinomas in the canine female, its use can help in the identification of certain types of mammary carcinoma more aggressive, providing prognostic value to the diagnosis.

In this work, in order to make comparisons with elastographic variables, we classified the tumors into three groups based on our previous study [26], in which survival was analyzed according to histological diagnosis, obtaining the best survival for complex carcinoma and the worst for anaplastic/inflammatory carcinoma, in agreement with other studies [40].

In human medicine, greater precision in the use of this elastographic modality is described, however, it should be taken into account that its use is limited to differentiating between benign and malignant tumors to provide complementary information in real time to conventional diagnosis, and to helping in the choice of surgical technique (regional vs. radical mastectomy).

The limitations of this elastographic modality are widely known, since it is an operator-dependent technique, with inter- and intraoperator differences that affect its reproducibility [22]. The results depend on the compression exerted by the person performing the test and this can affect the elasticity values and requires a learning curve. Our work was carried out by a single operator with more than five years of experience in performing this technique, therefore, no comparisons between observers have been made. Likewise, the tumor size can affect the precision of the test, since a lower accuracy has been described with a larger size of the lesion, the critical size being between 2.5 and 3 cm [22], so to reduce this bias as much as possible, only masses smaller than 3 cm were chosen. Ultimately, one of the most common causes of diagnostic error is calcium lesions such as fibroadenomas or carcinomas with necrosis or those with a lower consistency such as mucinous, which can give false positives and negatives, respectively [22]. Our results show how, in Group c, two of the malignant tumors with the lowest strain ratios presented areas of central necrosis, obtaining false negatives. We also observed a complex carcinoma (Group a) with higher strain ratios due to a high degree of fibrosis.

Therefore, it would be interesting to carry out more studies to analyze the inter-observer differences described for this diagnostic technique. Likewise, a greater knowledge of factors such as necrosis as well as calcifications or increased fibrosis in the mammary tissue, frequently associated with age, will allow for the diagnostic efficacy of this technique to be increased.

## 5. Conclusions

Strain elastography showed high accuracy in detecting intra- and peritumoral fibrosis using semi-quantitative methods even though this accuracy is limited in discriminating different types of carcinoma compared to other more advanced elastographic techniques. Strain elastography represents a useful diagnostic method to differentiate those malignant tumors with histological features that suggest greater aggressiveness. One of the greatest advantages of this diagnostic technique is that its performance requires little time and the interpretation of the results is quick, which allows for decision-making prior to surgery. In addition, it does not require large equipment, which facilitates its inclusion as an additional diagnostic tool in the clinic and is affordable for pet owners.

## Figures and Tables

**Table 1:** Histological diagnosis of malignant mammary neoplasms.

Histological Group	Histological Diagnosis	Percentage (n)
Group a	Complex carcinoma	32.14% (n = 18)
Group b	Mixed carcinoma	10.71% (n = 6)
	Ductal carcinoma/intraductal papillary carcinoma	14.29% (n = 8)
Group c	Simple carcinoma	21.43% (n = 12)
	Anaplastic carcinoma/inflammatory carcinoma	3.57% (n = 2)
	Carcinosarcoma/adenosquamous carcinoma/other special types of carcinomas	17.86% (n = 10)

**Table 2:** Visual score scale of malignant mammary carcinomas according to their histopathological features.

Visual Score Scale	Histological Variables				
	Histological Diagnosis			Intratumoral Fibrosis	
	Group A	Group B	Group C	Mild	High
Score 3	17.9% (n = 10)	14.3% (n = 8)	5.3% (n = 3)	17.9% (n = 10)	19.6% (n = 11)
Score 4	14.3% (n = 8)	10.7% (n = 6)	33.9% (n = 19)	17.9% (n = 10)	41.1% (n = 23)
Score 5	0.0% (n = 0)	0.0% (n = 0)	3.6% (n = 2)	1.8% (n = 1)	1.8% (n = 1)
*p* value	0.013			NS	

NS not significant.

**Table 3:** Intratumoral strain ratio of malignant mammary carcinomas according to their histopathological features.

Intratumoral Strain Ratio		Histological Diagnosis		Intratumoral Fibrosis	
	Group A	Group B	Group C	Mild	High
<4.25	19.6% (n = 11)	17.8% (n = 10)	17.8% (n = 10)	28.6% (n = 16)	26.8% (n = 15)
>4.25	12.5% (n = 7)	7.1% (n = 4)	25.0% (n = 14)	10.7% (n = 6)	33.9% (n = 19)
*p* value	0.018			0.03	

**Table 4:** Peritumoral strain ratio of malignant mammary carcinomas according to their histopathological features.

Peritumoral Strain Ratio	Peritumoral Fibrosis	
	Mild	High
<4.25	69.6% (n = 39)	5.4% (n = 3)
>4.25	1.8% (n = 1)	23.2% (n = 13)
*p* value	<0.001	

## Data Availability

The data presented in this study are available on request from the corresponding author.
